# Soy Whey Wastewater-Derived Sodium Alginate/Cellulose Composite Beads for Efficient Copper (II) Ion Adsorption: Performance and Mechanism

**DOI:** 10.3390/gels12060464

**Published:** 2026-05-26

**Authors:** Rui Li, Chang Xu, Qiannuo Gu, Xiaoyang Pan, Andong Qian, Xuning Leng

**Affiliations:** 1 School of Life Sciences, Jining Medical University, No. 669 Xueyuan Road, Donggang District, Rizhao 276826, China; xuchang@stu.mail.jnmc.edu.cn (C.X.); guqiannuo@stu.mail.jnmc.edu.cn (Q.G.); panxiaoyang@stu.mail.jnmc.edu.cn (X.P.); qiaandong@stu.mail.jnmc.edu.cn (A.Q.); 2 Municipal Science and Technology Innovation Service Center, Jining Road No. 369, Donggang District, Rizhao 276800, China

**Keywords:** adsorption, soy whey wastewater, sodium alginate, copper (II) ion

## Abstract

A sustainable alginate-based composite adsorbent was developed by valorizing soy whey wastewater for the efficient removal of copper (II) ions from aqueous solutions. Soy whey wastewater/sodium alginate/cellulose (SWWSAC) beads were fabricated via a controlled slow-release calcium ion cross-linking strategy. This strategy resulted in homogeneous gelation, effective encapsulation of wastewater-derived organics and the formation of a hierarchical mesoporous structure. Compared with pure sodium alginate (SA) and sodium alginate–cellulose (SAC) beads, the SWWSAC beads exhibited a significantly higher specific surface area (3.95 m^2^/g) and pore volume (0.021 cm^3^/g), thus having markedly enhanced copper (II) ion adsorption performance. Batch adsorption experiments demonstrate that the adsorption process was strongly dependent on solution pH, adsorbent dosage, contact time and initial metal concentration. Kinetic analysis indicates that the adsorption process followed a pseudo-second-order model, while equilibrium data were well described by the Langmuir isotherm, corresponding to monolayer chemisorption. Based on this isotherm, SWWSAC beads had a theoretical maximum adsorption capacity of 168.3 mg/g (25 °C), 190.8 mg/g (35 °C), and 204.4 mg/g (45 °C). Thermodynamic results reveal that the adsorption was spontaneous and endothermic. FTIR and XPS analyses confirm that copper (II) ion removal was governed by synergistic complexation involving carboxyl, hydroxyl, carbonyl, and protein-derived nitrogen-containing functional groups. Moreover, the SWWSAC beads had a copper (II) ion removal efficiency of (92.4 ± 0.4)% and retained 73.3% of their initial adsorption capacity after six regeneration cycles in actual electroplating wastewater treatment. In this process, the beads exhibited good anti-interference performance against coexisting cations and good structural stability. Therefore, this work demonstrates an effective and low-cost strategy for copper (II) ion removal while providing a value-added route for the sustainable utilization of soy whey wastewater.

## 1. Introduction

The extensive applications of copper in industrial and agricultural fields, such as smelting, electroplating, metal processing, and the production of fertilizers and pesticides, has increased global copper consumption considerably [1]. However, inadequate management of copper-containing waste has led to significant release of copper (II) ions into aquatic systems. These ions not only inhibit the growth of aquatic organisms and disrupt ecological equilibrium, but also accumulate through the food chain, posing substantial health risks to humans [2]. Chronic exposure to elevated copper levels may lead to hepatic and renal dysfunction, neurological disorders, and immune system impairments [3,4]. As a result, copper (II) ion pollution is now regarded as a critical global environmental issue [5]. To address this problem, several treatment techniques have been developed, including adsorption, membrane separation, and electrochemical methods [6,7,8]. Among these, adsorption is widely adopted due to its cost-effectiveness, operational simplicity, and high removal efficiency [1,9].

The effectiveness of adsorption essentially depends on the performance of the adsorbent material. In industrial wastewater treatment, conventional adsorbents—including activated carbon [10], ion-exchange resins [11], montmorillonite, and kaolin [12]—are widely used for removing copper (II) ions. However, these materials suffer from several drawbacks, including limited adsorption capacity, poor reusability, and a tendency to foul and contaminate. In recent years, sodium alginate–based adsorbents have attracted growing interest due to their promising performance in removing heavy metal ions from aqueous systems. Their appeal lies in their environmental friendliness, abundance of functional groups (e.g., hydroxyl and carboxyl), and high modifiability [13]. For example, Li et al. [14] fabricated mesoporous gel beads composed of nanocellulose, sodium alginate, and carboxymethyl-chitosan, reporting a copper (II) ion adsorption capacity of 169.94 mg/g. In another study, Liang et al. [15] developed an MXene/polyaniline/sodium alginate composite gel that achieved a markedly higher adsorption capacity of 255.81 mg/g. These results highlight the potential of sodium alginate–based composites as efficient adsorbents for copper (II) ion removal, especially when incorporating organic constituents.

Soy whey wastewater is generated as a byproduct during the production of soy protein isolate (SPI) through alkaline dissolution and acid precipitation [16]. It is characterized by high concentrations of organic constituents such as residual proteins, isoflavones, organic acids, and oligosaccharides [17]. In conventional SPI manufacturing processes, this effluent is typically discharged into municipal sewage systems for biochemical treatment [18]. Such practice not only results in the loss of nutritionally and functionally valuable compounds but also imposes a significant economic burden on producers through wastewater management costs. Previous research has established that copper (II) ions can form stable coordination complexes with specific amino acid residues present in proteins [19,20]. Furthermore, soy-derived isoflavones and oligosaccharides—which are abundant in hydroxyl functional groups—may facilitate copper (II) ion binding through direct chelation or by enhancing the protein-copper (II) interactions [21,22]. Capitalizing on these mechanistic insights, the present study will introduce a strategy for valorizing soy whey wastewater by incorporating it as a functional component within a composite adsorbent system based on sodium alginate and cellulose.

Calcium ion cross-linking is a widely adopted, cost-effective method for synthesizing sodium alginate-based adsorbents due to its simplicity [23]. In conventional methods, a sodium alginate solution is dripped into a calcium chloride solution, and this procedure leads to rapid gelation through ion exchange and the formation of solid hydrogel beads [24]. However, in systems containing soy whey proteins, high concentrations of calcium chloride may trigger protein precipitation via a salting-out effect before gelation, so that the effective encapsulation of protein constituents within the alginate matrix will be hindered. To overcome this limitation, we propose a slow-release ion cross-linking strategy for the synthesis of soy whey wastewater/sodium alginate/cellulose (SWWSAC) composite beads. This method will involve introducing a homogeneous mixture of soy whey wastewater, sodium alginate, cellulose, and finely dispersed calcium carbonate particles into an oil phase containing acetic acid. The gradual acid-induced dissolution of calcium carbonate will release calcium ions in a controlled manner. This manner will facilitate uniform cross-linking of sodium alginate and promote efficient encapsulation of soy whey components. Furthermore, the carbon dioxide generated during the reaction between calcium carbonate and acetic acid will contribute to the development of an interconnected porous structure within the beads. In this case, the available surface area and adsorption capacity will be increased.

To elucidate the roles of soy whey wastewater and cellulose in copper (II) ion adsorption, SWWSAC beads will be characterized using scanning electron microscopy (SEM), Fourier-transform infrared spectroscopy (FTIR), and nitrogen adsorption–desorption isotherms based on the Brunauer–Emmett–Teller (BET) method. Sodium alginate-cellulose (SAC) and pure sodium alginate (SA) beads will serve as reference materials. Subsequently, the adsorption capacities of all three adsorbents for copper (II) ions will be systematically compared. Following this analysis, the adsorption performance of SWWSAC beads under varying environmental conditions will be evaluated. To achieve deeper insights into the adsorption mechanism, kinetic, thermodynamic, FTIR, and X-ray photoelectron spectroscopy (XPS) analyses will be conducted. Finally, SWWSAC beads will be used to remove copper (II) ions from actual electroplating wastewater, and their removal efficiency and reusability will be assessed. All these investigations aim to develop a high-performance, recyclable copper (II) ion adsorbent while promoting an environmentally sustainable approach to valorizing soy whey wastewater.

## 2. Results and Discussion

### 2.1. Characterization Results

#### 2.1.1. SEM-EDS Analysis

**Figure 1:**
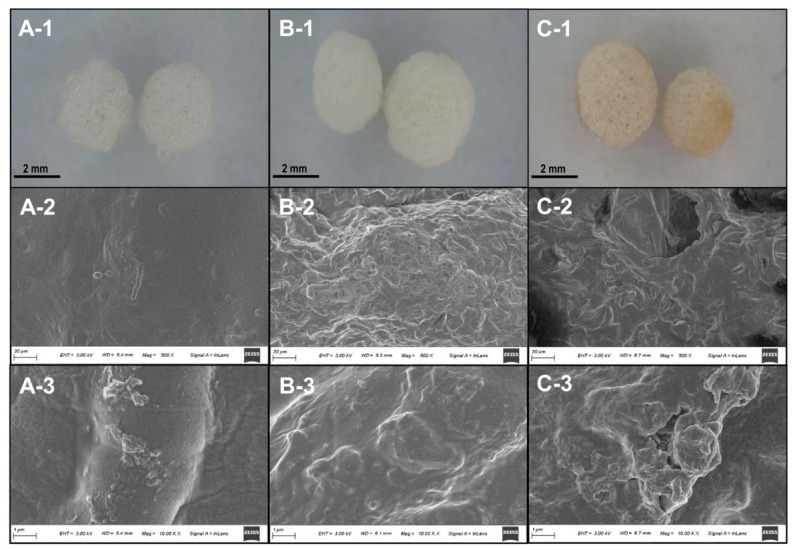
Optical micrographs of SA (**A-1**), SAC (**B-1**), and SWWSAC (**C-1**) beads and SEM images of SA beads at magnifications of ×500 (**A-2**) and ×10,000 (**A-3**), SAC beads at magnifications of ×500 (**B-2**) and ×10,000 (**B-3**), and SWWSAC beads at magnifications of ×500 (**C-2**) and ×10,000 (**C-3**).

**Figure 2:**
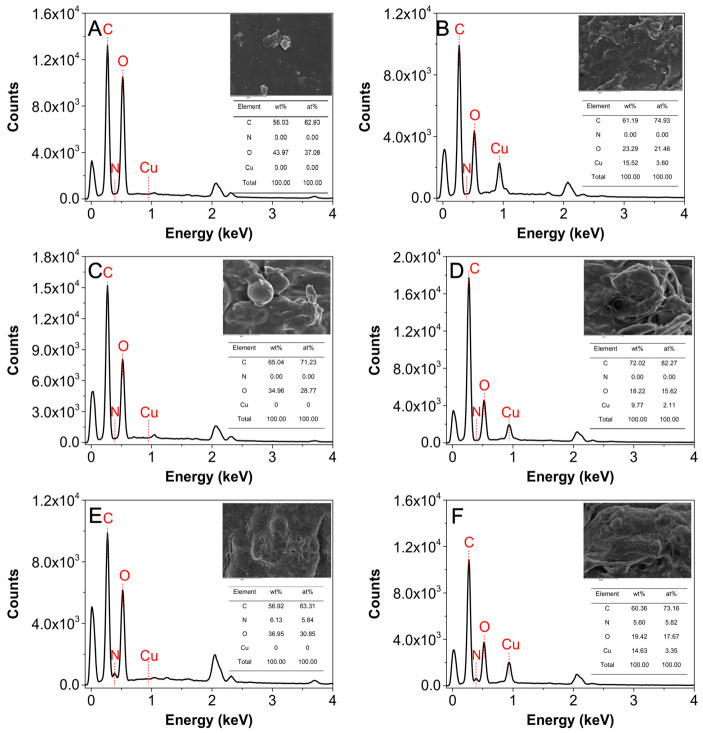
EDS spectra and corresponding elemental composition of SA beads (**A**,**B**), SAC beads (**C**,**D**) and SWWSAC (**E**,**F**) beads before and after copper (II) ion adsorption.

**Figure 3:**
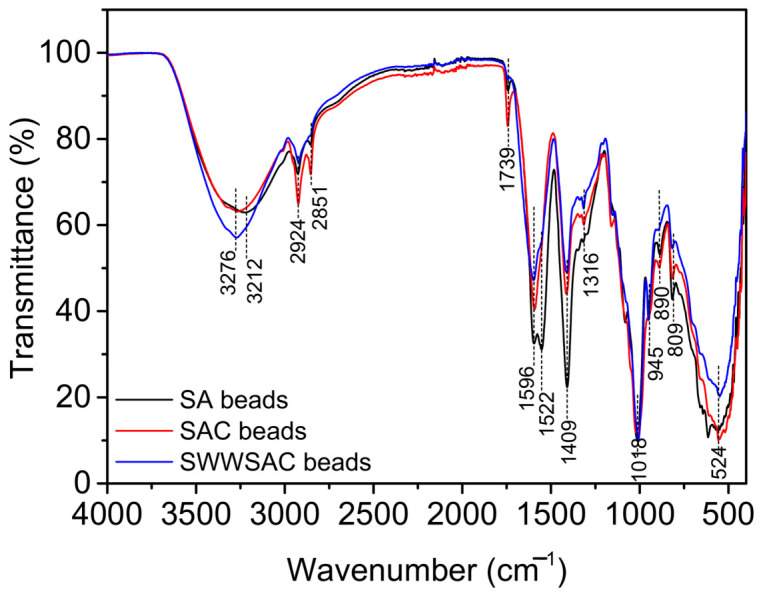
FTIR spectra of SA, SAC, and SWWSAC beads.

The macroscopic and microscopic morphologies of SA, SAC and SWWSAC beads were characterized, as shown in Figure 1. Macroscopically, the beads (Figure 1(A-1,B-1,C-1)) exhibited diameters of 3.15 ± 0.15 mm (SA), 3.34 ± 0.12 mm (SAC), and 3.43 ± 0.17 mm (SWWSAC), respectively. Statistical analysis confirms that these dimensional differences were significant (*p* ≤ 0.05). The increase in bead diameter following the incorporation of cellulose and soy whey wastewater components suggests that these additives influenced the cross-linking dynamics between sodium alginate and calcium ions, ultimately leading to greater bead volume [25]. In terms of visual appearance, the transparency of the SAC beads decreased relative to the SA beads due to the presence of cellulose. Meanwhile, the SWWSAC beads developed a distinct light-yellow hue, similar to the color of the soy whey wastewater. This phenomenon indicates the successful integration of wastewater constituents into the bead matrix. Surface morphology analysis further revealed significant textural differences among the bead types. The SA beads displayed a relatively smooth surface with sporadic island-like protrusions (Figure 1(A-2,A-3)). In contrast, the SAC beads showed increased surface roughness, and this phenomenon was more pronounced in the SWWSAC beads (Figure 1(B-2,B-3,C-2,C-3)). Particularly, the addition of soy whey wastewater resulted in the formation of visible pores on the bead surface. These observations collectively demonstrate that incorporating cellulose and soy whey wastewater promoted the development of a more porous and architecturally complex cross-linked network within the beads.

The EDS analysis revealed the elemental composition and distribution of SA, SAC and SWWSAC beads before and after copper (II) ion adsorption, as presented in Figure 2. Comparative analysis of the spectra (Figure 2A,C) indicates a higher relative carbon content and a lower oxygen content in SAC beads compared to SA beads. This difference was attributed to the lower carbon-to-oxygen ratio of cellulose relative to sodium alginate. This finding confirms the successful incorporation of cellulose into the sodium alginate–calcium ion network. In the SWWSAC beads (Figure 2E), the detection of nitrogen, accompanied by a reduced relative carbon content compared to SAC beads, suggests the integration of proteins originating from the soy whey wastewater. Furthermore, the relative oxygen content in SWWSAC beads was higher than in SAC beads due to the introduction of oxygen-rich organic compounds from the wastewater, such as oligosaccharides and organic acids. After copper (II) ion adsorption (Figure 2B,D,F), copper was identified on all three bead types. The highest relative copper content was observed on SA beads, while SWWSAC beads exhibited a higher adsorption capacity than SAC beads. These results demonstrate that the incorporation of cellulose alone is unfavorable for copper (II) ion adsorption because of its weak binding affinity for copper (II) ions [26]. In contrast, the organic compounds derived from soy whey wastewater significantly enhanced copper (II) ion adsorption. Thus, the overall binding capacity of the SWWSAC beads was enhanced.

#### 2.1.2. FTIR Analysis

FTIR spectroscopy was employed to characterize the functional groups present in the SA, SAC, and SWWSAC beads, as shown in Figure 3. Characteristic absorption peaks were consistently identified in all three samples at 2924, 2851, 1739, 1596, 1409, 1316, 1018, 890, 809, and 524 cm^−1^. These bands were assigned to the stretching vibrations of C–H (from –CH_2_/CH_3_ groups), C=O (from –COOH/–COO^−^ groups), -COOH, C–O–C (from the sugar ring skeleton), and Ca–O bonds [27]. The presence of these functional groups confirms the successful formation of a sodium alginate–calcium ion cross-linked network in the three adsorbent types.

A comparative analysis of the SA and SAC bead spectra revealed that the incorporation of cellulose induced a blue shift in the –OH stretching vibration peak from 3212 cm^−1^ to 3276 cm^−1^. This shift suggests that cellulose weakened the intrinsic hydrogen-bonding interactions within the SAC beads, resulting in a looser alginate–calcium ion network. This observation contrasted with findings reported by Deng et al. [28] that cellulose particles were shown to enhance hydrogen bonding with sodium alginate. Additionally, the increased intensities of the peaks at 2924, 2851, and 1739 cm^−1^ indicate the successful encapsulation of cellulose within the SAC matrix. These spectral changes also imply the formation of new hydrogen bonds between cellulose and sodium alginate, which intensified the C=O stretching vibrations. However, the blue shift of the –OH peak indicates that these newly formed bonds appeared weaker than those among sodium alginate molecules themselves [29]. Furthermore, cellulose incorporation reduced the intensities of the peaks at 1596, 1522, 1409, 890, and 809 cm^−1^. This result could be attributed to both the formation of new hydrogen bonds and a partial disruption of the original alginate–calcium ion cross-linked network.

Compared to the SAC beads, the FTIR spectrum of the SWWSAC beads exhibited a more obvious and sharper peak at 3276 cm^−1^. This finding was attributed to the vibration of N-H from –NH_2_ or C(=O)-NH- of soy whey proteins. Moreover, the SWWSAC spectrum showed decreased intensities at 2924, 2851, 1739, 1596, 1409, 890, 809, and 524 cm^−1^, while the peak at 945 cm^−1^ increased in intensity. These phenomena suggest that sodium alginate and cellulose interact with the organic compounds in soy whey wastewater. Specifically, proteins, oligosaccharides, or organic acids in the wastewater may competitively bind to hydroxyl groups from sodium alginate and cellulose to form stronger hydrogen bonds [30]. In this case, the existing hydrogen-bonding network between sodium alginate chains and sodium alginate–cellulose was disrupted. These newly formed hydrogen bond network or protein- sodium alginate complexes might restrict the vibrations of CH_2_, C=O, -COOH and C–O–C bonds [31]. As a result, the intensities at 2924, 2851, 1739, 1596, 1409, 890 and 809 cm^−1^ were reduced. In addition, proteins in the wastewater may interact with carboxyl groups of sodium alginate, competing with alginate–calcium ions, thereby leading to a reduced intensity at 524 cm^−1^. The increasing intensity at 945 cm^−1^ indicates the interaction between –COOH from sodium alginate and –NH_3_^+^ from soy whey proteins [32]. The interaction between proteins and sodium alginate could explain the slightly lower relative content of copper in the small surface area of SWWSAC beads than that in the small surface area of SA beads.

#### 2.1.3. BET Analysis

**Figure 4:**
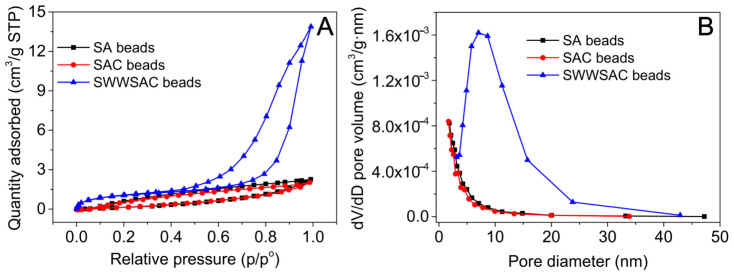
Nitrogen adsorption–desorption isotherms (**A**) and pore size distribution plots (**B**) of SA, SAC, and SWWSAC beads.

The specific surface area and pore-size distribution of the SA beads, influenced by the incorporation of cellulose and soy whey wastewater components, were characterized by nitrogen adsorption–desorption analysis (Figure 4). As illustrated in the isotherm plots (Figure 4A), both the SA and SAC beads exhibited a slow increase in nitrogen adsorption capacity, reaching final values of 2.26 cm^3^/g STP and 2.03 cm^3^/g STP, respectively, across the entire relative pressure (p/p^0^) range from 0 to 1.0. In marked contrast, the SWWSAC beads showed a rapid surge in adsorption capacity, particularly in the high-pressure region (p/p^0^ > 0.8), attaining a capacity of 13.88 cm^3^/g STP. This profile is indicative of a Type IV isotherm, which is characteristic of mesoporous materials [33]. The corresponding specific surface areas, summarized in Table 1, were 1.18 m^2^/g for SA beads, 2.10 m^2^/g for SAC beads, and 3.95 m^2^/g for SWWSAC beads. These quantitative results confirm that the modification with soy whey wastewater was particularly effective in creating a more porous structure with a substantially larger specific surface area.

The pore size distribution profiles further elucidated the structural differences between the adsorbents (Figure 4B). The SA and SAC beads displayed similar curves, with pore diameters predominantly within the 1.84–10 nm range. Conversely, the SWWSAC beads exhibited a distinct, sharp peak in the mesoporous region (5–25 nm). Consistent with this observation, the SWWSAC beads possessed a significantly larger total pore volume (0.021 cm^3^/g) and average pore diameter (9.81 nm) compared to the SA (0.0035 cm^3^/g, 4.03 nm) and SAC beads (0.0031 cm^3^/g, 3.87 nm). The combined data from the textural analysis demonstrate that the addition of cellulose moderately increased the specific surface area of the SA matrix, while the incorporation of soy whey wastewater components profoundly enhanced both the specific surface area and the pore volume by introducing a well-defined mesoporous structure. This optimized pore architecture was conducive to the enhanced adsorption of copper (II) ions. Overall, these findings were in full agreement with the morphological features observed via SEM (Figure 1).

### 2.2. Adsorption Experiments

#### 2.2.1. Effect of Type of Beads

**Figure 5:**
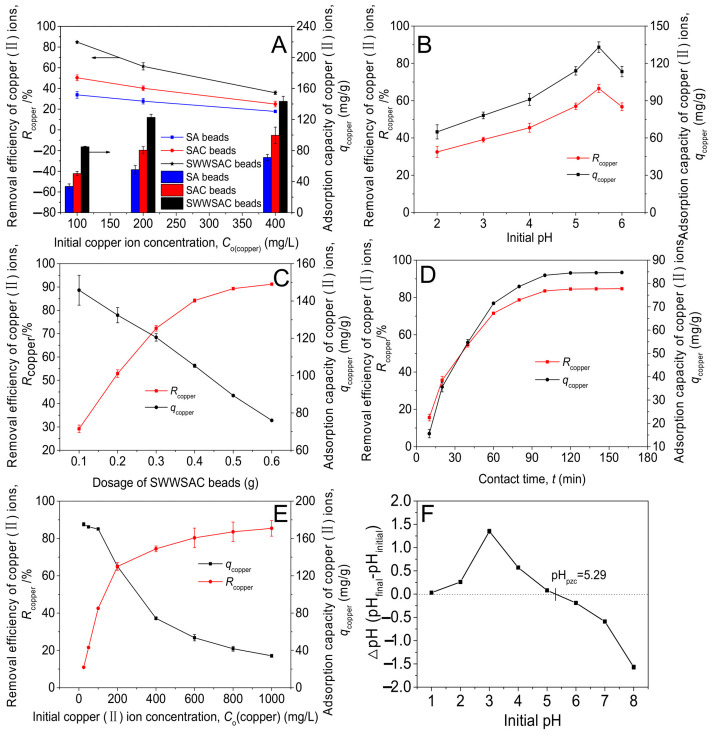
Factors influencing the removal efficiency and adsorption capacity of copper(II) ions using SWWSAC beads: (**A**) adsorbent type, (**B**) solution pH, (**C**) adsorbent dosage, (**D**) contact time, and (**E**) initial copper (II) concentration and the isoelectric point of the SWWSAC beads via the pH drift method (**F**).

Based on the characterization results (Section 2.1), the adsorption performance of SA, SAC, and SWWSAC beads for copper (II) ions was compared under controlled conditions (initial concentrations: 100, 200, 400 mg/L; bead dosage: 0.25 g; pH 5.0). According to Figure 5A, SAC and SWWSAC beads exhibited significantly higher removal efficiency and adsorption capacity than SA beads across all concentrations, with SWWSAC performing best. The performance gap between SAC and SWWSAC was more significant than that between SA and SAC. Although cellulose incorporation increased the specific surface area of SA beads (Table 1), it reduced the adsorption capacity on the infinitesimal area (Figure 2). In contrast, adding soy whey wastewater further enhanced specific surface area, pore size, and pore volume (Table 1), thus improving mass transfer of copper (II) ions. Furthermore, SWWSAC beads exhibited a slightly lower adsorption capacity on the infinitesimal area compared to SA beads. These observations suggest that soy whey wastewater was more effective than cellulose in enhancing copper (II) adsorption by SA beads. Thus, SWWSAC was selected as the preferred adsorbent for subsequent studies.

#### 2.2.2. Effect of Initial pH

The adsorption performance of sodium alginate-based adsorbents is strongly influenced by pH, as it affects surface charge properties [34]. To systematically evaluate this relationship, copper (II) ion adsorption on SWWSAC beads was investigated under controlled conditions: initial copper (II) concentration of 200 mg/L, adsorbent dosage of 0.25 g, and pH range of 2.0–6.0. As depicted in Figure 5B, the removal efficiency increased from (32.5 ± 2.9)% to (66.5 ± 2.1)% as pH rose from 2.0 to 5.5, but decreased to (56.7 ± 2.1)% at pH 6.0. A similar trend was observed for adsorption capacity. The isoelectric point of SWWSAC beads was determined to be pH 5.29 (Figure 5F). Below this value, the bead surface carried a net positive charge, leading to electrostatic repulsion of copper (II) ions. As pH approached the isoelectric point, the surface charge was gradually neutralized, reducing repulsive interactions and improving adsorption. Above pH 5.29, the surface had a net negative charge. This would theoretically enhance adsorption through electrostatic attraction. Furthermore, Figure S1 shows that the relative content of free copper (II) ions had just a slight decrease in the pH range from 2.0 to 5.5. As a consequence, the adsorption of copper (II) ions was enhanced in this pH range. However, both removal efficiency and adsorption capacity decreased at pH 6.0. It was attributed to the extensive formation of copper (II) monohydroxide ions at this pH (Figure S1). Their formation reduced the effective positive charge of aqueous copper species and decreased the availability of strongly adsorbable free copper (II) ions. Therefore, the electrostatic attraction to negatively charged sites and the overall copper (II) adsorption efficiency were reduced.

#### 2.2.3. Effect of Dosage of SWWSAC Beads

Adsorbent dosage critically determines the economic feasibility of heavy metal removal processes. To evaluate this for SWWSAC beads, we examined their dosage-dependent performance in copper (II) ion adsorption under fixed conditions: initial copper (II) concentration of 200 mg/L, pH 5.5, and bead dosages ranging from 0.1 to 0.6 g. As shown in Figure 5C, the removal efficiency increased markedly from (29.2 ± 1.6)% to (84.2 ± 0.7)% with rising dosage, eventually plateauing near (91.2 ± 0.4)% at 0.6 g. Conversely, the adsorption capacity decreased sharply from 145.8 ± 8.0 mg/g to 76.0 ± 0.4 mg/g over the same range. This trend was consistent with that of Zhu et al. [35] for the adsorption of sodium dodecyl benzene sulfonate. This inverse relationship stemmed from the interplay between active site availability and metal ion concentration. At lower dosages, the limited adsorbent quantity allowed full utilization of active sites per unit mass to maximize capacity. As dosage increased, the total active sites surpassed the stoichiometric requirement for the fixed copper (II) ion mass. This led to saturation in removal efficiency. Consequently, the constant amount of copper (II) ions became distributed across excess sites and thereby reduced the adsorption capacity per gram of adsorbent.

#### 2.2.4. Effect of Contact Time

Contact time critically influences the attainment of adsorption equilibrium by regulating the interaction time between the adsorbent and the solute. To evaluate this effect on copper(II) ion adsorption by SWWSAC beads, time-dependent experiments were conducted, with removal efficiency and adsorption capacity serving as key indicators (Figure 5D). Both parameters increased rapidly during the initial stages (0 to 60 min) before plateauing at equilibrium. This trend was attributed to the high availability of vacant active sites at early phases, which promoted swift uptake of copper (II) ions [36]. As adsorption progressed (60 to 100 min), site saturation increased so that diffusion resistance was elevated and the adsorption rate was slowed. Finally, equilibrium was achieved when all accessible sites were occupied (100 to 160 min). In this case, the removal efficiency and adsorption capacity no longer changed significantly.

#### 2.2.5. Effect of Initial Copper (II) Ion Concentration

The initial concentration of copper (II) ions significantly influences the adsorption performance of SWWSAC beads by altering the mass transfer driving force and the saturation of active sites. As shown in Figure 5E, when the initial copper (II) ion concentration increased from 25 mg/L to 1000 mg/L, the removal efficiency decreased from (87.6 ± 0.9)% to (17.1 ± 0.8)%, while the adsorption capacity increased sharply from 21.9 ± 0.2 mg/g to 130 ± 3.9 mg/g and then slowly to 170.9 ± 8.4 mg/g. These trends are consistent with previous reports [37]. At low concentrations (25–100 mg/L), the abundance of vacant active sites on the SWWSAC surface enabled efficient binding of copper (II) ions. The increasing concentration gradient strengthened the mass transfer driving force and thereby promoted the migration of ions to the adsorbent surface. This led to a marked increase in adsorption capacity with only a modest decrease in removal efficiency. At higher concentrations (>100 mg/L), the active sites approached saturation, so further uptake was limited and hence a rapid decline in removal efficiency was caused. However, owing to the greater total quantity of copper (II) ions in the solution, the adsorption capacity continued to increase, but at a diminished rate.

### 2.3. Adsorption Kinetics, Isotherms and Thermodynamics

#### 2.3.1. Adsorption Kinetics

**Figure 6:**
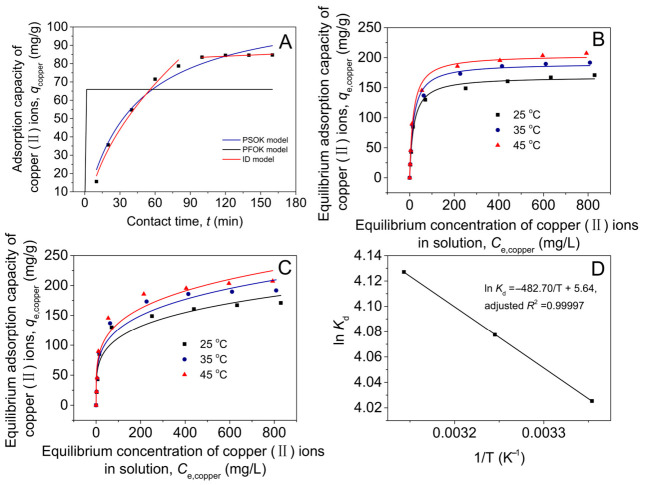
Kinetic and isotherm models for the adsorption of copper (II) ions onto SWWSAC beads. (**A**) Fitting of adsorption capacity versus contact time data using the pseudo-first-order kinetic (PFOK), pseudo-second-order kinetic (PSOK), and intraparticle diffusion (ID) models. (**B**,**C**) Fitting of equilibrium adsorption capacity versus equilibrium solution concentration data using the (**B**) Langmuir and (**C**) Freundlich isotherm models at 25, 35, and 45 °C. (**D**) Linear relationship between ln *K*_d_ and 1/T for thermodynamic parameter estimation.

The adsorption kinetics of copper (II) ions onto SWWSAC beads were evaluated by fitting the time-dependent adsorption capacity data (Figure 5D) to pseudo-first-order kinetics (PFOK), pseudo-second-order kinetics (PSOK), and intraparticle diffusion (ID) models. The fitting curves and corresponding parameters are presented in Figure 6A and Table 2, respectively. As presented, the PFOK model yielded a relatively high *q*_e,copper_ error of 13.7%, a negative adjusted R^2^, and an extremely high *χ*^2^ value. This result indicates that the model failed to describe the kinetic data and performed worse than a simple mean-value fit. In contrast, the PSOK model showed a lower *q*_e,copper_ error of 5.7%, a high adjusted R^2^ value, and a substantially lower *χ*^2^ value. Considering both statistical goodness-of-fit and parameter validity, the PSOK model was deemed the most appropriate. It is suggested that the adsorption process involves both physical and chemical interactions [38]. The ID model was also applied to identify whether intraparticle diffusion acts as the rate-limiting step. It had a high adjusted R^2^ value and a lower *χ*^2^ value, thus confirming its ability to describe the adsorption kinetics. As shown in Figure 6A, the ID model aligned well with experimental observations within the 10–80 min timeframe. However, the nonzero intercept (Θ = −15.9 mg/g) indicates that the diffusion plot did not pass through the origin and implied additional influence from boundary layer (film) diffusion as a co-controlling mechanism in the overall adsorption kinetics [39].

#### 2.3.2. Adsorption Isotherms

Based on the data presented in Figure 5E, the adsorption isotherms of copper (II) ions on SWWSAC beads were determined at 25 °C by measuring the equilibrium adsorption capacity and the residual copper(II) ion concentration. Following the same methodology, the adsorption isotherms at 35 °C and 45 °C were also obtained. The resulting isotherm data were fitted to both the Langmuir and the Freundlich adsorption models, as illustrated in Figure 6B,C and Table 3. At all investigated temperatures, the Langmuir model exhibits substantially higher adjusted *R*^2^ values (0.9909–0.9933) and much lower *χ*^2^ values (32.7–54.0) compared to the Freundlich model. Thus, the Langmuir adsorption model was more appropriate for describing the adsorption behavior of copper (II) ions onto SWWSAC beads. According to the Langmuir model fitting results, the maximum adsorption capacities of SWWSAC beads at 25 °C, 35 °C, and 45 °C are 168.3 mg/g, 190.8 mg/g, and 204.4 mg/g, respectively.

Table 4 summarizes the maximum adsorption capacities of copper (II) ions by various sodium alginate-based adsorbents as reported in previous studies [14,40,41,42,43,44,45,46,47,48]. Among these materials, the SWWSAC beads exhibited a comparatively higher adsorption capacity than most of the adsorbents listed. However, their capacity was lower than that of several advanced composites, including ZIF-8@ sodium alginate composite hydrogels [45], sodium alginate-Mg(OH)_2_ hydrogels [46] and chitosan/carboxymethyl cellulose/sodium alginate magnetic composite hydrogels [48]. It is noteworthy that the synthesis cost of SWWSAC beads was significantly lower than that of these three high-performance adsorbents. Therefore, SWWSAC beads could be regarded as a cost-effective alternative for the removal of copper (II) ions from aqueous solutions.

#### 2.3.3. Adsorption Thermodynamics

The influence of temperature on the adsorption of copper (II) ions by SWWSAC beads was investigated to elucidate the thermodynamic characteristics of the process. As summarized in Table 3, elevating the temperature from 25 °C to 35 °C resulted in a moderate increase in the maximum adsorption capacity from 168.3 mg/g to 204.4 mg/g. Concurrently, the Langmuir constant (*K*_L_) rose from 0.056 L/mg to 0.062 L/mg. This result reflects an enhanced affinity between the adsorbent and copper (II) ions at higher temperatures. To quantitatively assess the thermodynamic behavior, the van’t Hoff equation was applied to the linear relationship between ln *K*_d_ and 1/T (Figure 6D) to achieve ΔH and ΔS values. The ΔG was subsequently derived using the Gibbs equation. All the calculated thermodynamic parameters are shown in Table 5. The negative ΔG values confirm the spontaneity of the adsorption process across the studied temperature range. Furthermore, the positive ΔH and ΔS values indicate that the adsorption is endothermic and accompanied by an increase in randomness at the solid–liquid interface [49]. This rise in disorder might be attributed to the release of water molecules from the hydration shells of copper (II) ions during adsorption [50].

### 2.4. Adsorption Mechanisms

**Figure 7:**
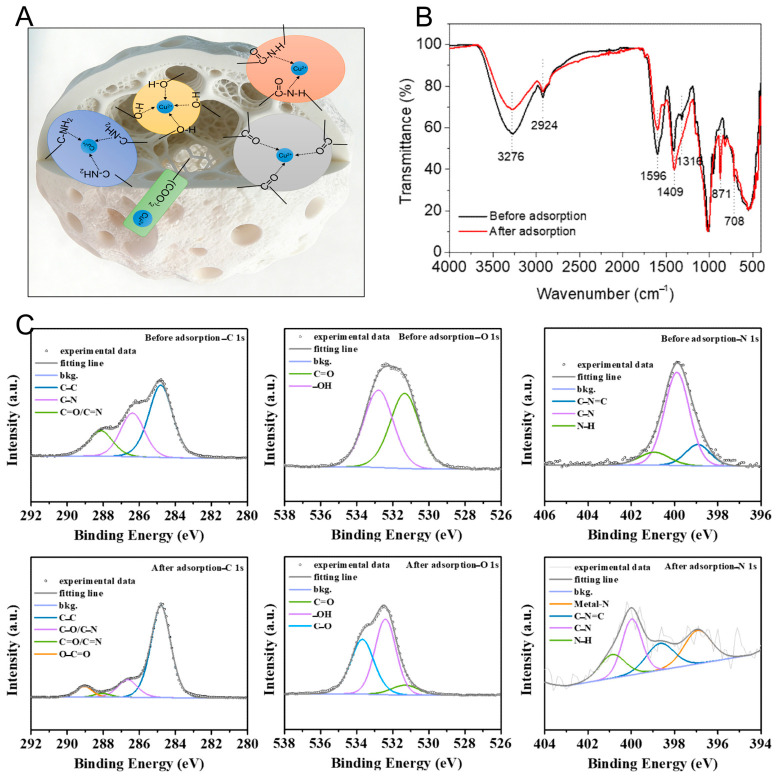
Schematic diagram of the mechanisms of copper (II) ion adsorption by SWWSAC beads (**A**), FTIR spectra of SWWSAC beads before and after copper (II) ion adsorption (**B**) and XPS spectra of SWWSAC beads before and after copper (II) ion adsorption: C 1s, O 1s, and N 1s spectra before adsorption; C 1s, O 1s, and N 1s spectra after copper (II) ion adsorption (**C**).

To explore the mechanisms for the adsorption of copper (II) ions onto SWWSAC beads, we characterized the beads before and after adsorption using FTIR and XPS. The characterization results are presented in Figure 7B and Figure 7C, respectively.

Figure 7B shows that after adsorption of copper (II) ions, the FTIR spectrum of SWWSAC beads presents weakening of peaks at 3276, 2924, 1596, and 1316 cm^−1^, intensification of the peak at 1405 cm^−1^, and the emergence of new peaks at 871 and 708 cm^−1^. The decrease in peak intensity at 3276 cm^−1^, corresponding to the O-H stretching vibration of -OH, indicates that -OH groups were involved in coordination with copper (II) ions [51]. The reduced intensity of the C-H stretching vibration at 2924 cm^−1^ resulted from restricted vibrational freedom of aliphatic chains in proteins from soybean wastewater owing to their encapsulation or environmental changes within the bead matrix [52]. The significant decrease in the asymmetric stretching vibration of -COO^−^ at 1596 cm^−1^ coupled with the increase in the symmetric stretching vibration at 1405 cm^−1^ provides direct evidence that -COO^−^ served as the primary binding site for copper (II) ion adsorption [53]. The binding between them through ion exchange modified the electron cloud distribution and vibrational symmetry of -COO^−^, thus resulting in their peak intensity changes. The decrease in peak intensity at 1316 cm^−1^, associated with -OH or C-O vibrations, further supported the involvement of these functional groups in the copper (II) ion adsorption. The appearance of new peaks at 871 and 708 cm^−1^ was attributable to stretching vibrations of the newly formed Cu-O bonds [54]. This finding was a direct spectroscopic evidence of stable chelate structure formation between copper (II) ions and the bead matrix.

From Figure 7C, the XPS spectra of SWWSAC exhibited significant changes before and after copper (II) ion adsorption. Specifically, three peaks for C-C (284.8 eV), C-O/C-N (286.38 eV), and C=O/N-C=O (288.14 eV) appeared in the C 1s spectrum. These results reflect the basic carbon framework and functional groups of cellulose, sodium alginate, and proteins. After adsorption, a new O-C=O peak at a higher binding energy (~289.5 eV) emerged after adsorption. It is indicated that the -COO^−^ groups interacted with copper (II) ions, and this interaction led to a decrease in the electron cloud density of the connected carbon atoms and an increase in binding energy [55]. Furthermore, the C-O/C-N peak shifted from 286.38 eV to 286.65 eV, and the C=O/C=N peak from 288.14 eV to 288.05 eV. The O 1s spectra before and after adsorption illustrate that the relative atomic peak area for C=O (531.35 eV) decreased from 49.42% to 8.46% and the new peak for C-O (533.68 eV) appeared with a relative area of 41.29%. Meanwhile, the peak for -OH shifted from 532.8 eV to 532.4 eV. These findings demonstrate that oxygen atoms in -OH and C=O groups and nitrogen-containing functional groups participated in complexation with copper (II) ions [56].

The most critical evidence for the involvement of proteins in soybean wastewater in copper (II) ion adsorption was derived from the changes in the N 1s spectrum. After adsorption, the nitrogen signal originating from proteins exhibited a significantly reduced signal-to-noise ratio. Concurrently, a new peak attributed to Metal-N coordination appeared at a lower binding energy (398.0 eV). Furthermore, the peak area for C-N (399.89 eV) decreased from 71.65% to 32.83% while the peak areas for C-N=C (398.92 eV) and N-H (400.94 eV) slightly increased. However, the C-N=C bonds might belong to other N-containing compounds rather than proteins in soy whey wastewater. These findings directly confirm the formation of stable N–Cu coordination bonds between N-containing functional groups within the proteins (mainly amino and amide groups) and copper (II) ions [57].

In summary, the adsorption mechanism of copper (II) ions onto the SWWSAC beads involved a synergistic chemical complexation process mediated by multiple functional groups, including carboxyl, hydroxyl, carbonyl, amino, and amide groups. Based on these results, a schematic diagram of the mechanisms of copper (II) ion adsorption by SWWSAC beads was described in Figure 7A.

### 2.5. Application of SWWSAC Beads in Removing Copper (II) Ions from Actual Electroplating Wastewater

**Figure 8:**
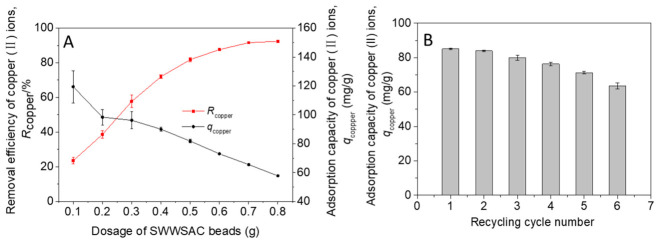
Effects of bead dosage on removal efficiency and adsorption capacity (**A**) and Variation of adsorption capacity with recycling cycle number (**B**) during the removal of copper (II) ions from actual electroplating wastewater using SWWSAC beads.

To explore the practical application value of SWWSAC beads, we used them to remove copper (II) ions from the actual electroplating wastewater and studied the effects of their dosage on the removal efficiency and adsorption capacity of copper (II) ions. The adsorption experiments were carried out in a batch model at wastewater volume 250 mL, initial pH of 5.5, bead dosage of 0.1–0.8 g, contact time of 120 min, and temperature of 25 °C. The results in Figure 8A show that the removal efficiency increased from (23.5 ± 1.8)% to (92.4 ± 0.4)% while the adsorption capacity decreased from 119.4 ± 11.2 mg/g to 57.7 ± 0.2 mg/g as dosage of SWWSAC beads increased. Compared with the results in Figure 5C, SWWSAC beads had lower removal efficiency and adsorption capacity for actual electroplating wastewater than for the prepared copper (II) ion wastewater. This resulted from competition between cations in the actual electroplating wastewater—particularly divalent heavy-metal ions—and copper (II) ions for the available adsorption sites on the adsorbent [1,5].

At a bead dosage of 0.8 g, we further explored the competitive adsorption of copper (II) ions with other cations in the electroplating wastewater using SWWSAC beads as the adsorbent. Corresponding data are presented in Table 6. The copper (II) ion removal efficiency and adsorption capacity far exceeded those for other heavy metals (cadmium (II) ion, lead(II)ion, zinc (II)ion and nickel (II)ion, 13.7–26.0%) and alkali/alkaline earth metals (sodium (I) ion, potassium (I) ion and calcium (II) ion, 7.6–42.1%). The distribution coefficient for copper (II) ions reached 3.15 L/g. It was much higher than those for competing cations, and their selectivity coefficients relative to copper (II) ion were ranged from 14.3 to 105. These findings confirmed strong preferential adsorption of SWWSAC beads toward copper (II) ions. This exceptional selectivity is attributed to the specific coordination interactions between copper (II) ion and the functional groups on SWWSAC beads. In addition, the wastewater pH remained stable at 5.1 ± 0.1 after adsorption, avoiding metal hydroxide precipitation. These results demonstrate that SWWSAC beads were highly effective and robust for selective copper (II) removal from complex electroplating wastewater matrices.

The recyclability of an adsorbent economically determines its industrialized usage prospects [14]. Therefore, this section explored the regenerability of SWWSAC beads using 0.1 mol/L HCl as the eluent. The copper (II) ions-loaded (II) were obtained from prior adsorption experiments at a bead dosage of 0.2 g. The resultant data are visualized in Figure 8B. After six consecutive reuse cycles, SWWSAC beads maintained an average copper (II) ion adsorption capacity of 72.1 mg/g—equivalent to 73.3% of their initial mean capacity. Furthermore, we compared the FTIR spectra of SWWSAC beads before copper (II) ion adsorption and after the 6th recycling cycle in Figure S2. It is revealed that the characteristic peaks at 3276, 1596, 1400, and 1314 cm^−1^ maintained their original positions but altered in intensity. Additionally, a new peak was observed at 869 cm^−1^. Compared to the FTIR spectra of SWWSAC beads after copper (II) ion adsorption in Figure 7B, these changes resulted from the interactions of copper (II) ions with functional groups on the adsorbent. Thus, the main skeleton of the beads remained stable, while the microenvironment of functional groups underwent persistent changes due to irreversible coordination or group consumption/loss. As a result, the adsorption capacity of SWWSAC beads decreased by 26.7% after six reuse cycles. Overall, these findings highlighted the effectiveness of SWWSAC beads for copper (II) ion removal from actual wastewater.

## 3. Conclusions

In this work, soy whey wastewater was successfully upcycled into a high-performance adsorbent, i.e., SWWSAC beads, through its integration with sodium alginate and cellulose using a controlled slow-release calcium ion cross-linking strategy. Compared to SA and SAC beads, the introduction of wastewater-derived organic components significantly altered the beads’ microstructure, leading to increased surface area (3.95 m^2^/g), enlarged mesopores (0.021 cm^3^/g), and enhanced accessibility of active sites. As a result, SWWSAC beads exhibited substantially increased copper (II) ion adsorption performance with a maximum adsorption capacity of up to 168.3–204.4 mg/g at 25–45 °C. Adsorption kinetics and isotherm analyses revealed that the copper (II) ion adsorption process was dominated by chemisorption and followed Langmuir-type monolayer adsorption behavior. Thermodynamic evaluation confirmed that the adsorption was spontaneous and endothermic. Spectroscopic investigations demonstrated that copper (II) ions were immobilized via synergistic coordination interactions involving carboxyl and hydroxyl groups from sodium alginate and cellulose, as well as amino and amide groups from soy whey proteins. In addition, the SWWSAC beads showed a good copper (II) ion removal ability from actual electroplating wastewater with a high removal efficiency of (92.4 ± 0.4)%, and a good anti-interference performance toward coexisting ions. After six adsorption–desorption cycles, the beads retained 73.3% of their initial adsorption capacity, and their main skeleton remained stable, indicating excellent reusability and structural stability. Overall, this study provides a sustainable and economically viable approach for the removal and recycling of copper (II) ions from soy whey wastewater, with strong potential for practical wastewater treatment applications.

## 4. Materials and Methods

### 4.1. Materials

Defatted soybean meal was procured from Hubei Xinghengye Technology Co., Ltd. (Wuhan, China). Actual electroplating wastewater was obtained from Wulian Wansheng Electroplating Industrial Co., Ltd., Rizhao, China, and its composition is listed in Table 7. Sodium alginate (chemical grade), α-cellulose (particle size ≤ 25 μm), acetic acid (analytical grade), dimethyl sulfoxide (analytical grade), absolute ethanol (analytical grade), and calcium carbonate (analytical grade) were sourced from Aladdin Biochemical Technology Co. Ltd., Shanghai, China, Sinopharm Chemical Reagent Co. Ltd., Shanghai, China, and Tianjin Beilian Fine Chemicals Development Co. Ltd., Tianjin, China, respectively. Corn germ oil was supplied by Zhongyi Huihai Grain and Oil Co., Ltd., Zhengzhou, China. Ultrapure water with a resistivity of 18.25 MΩ·cm was generated using a UPR-II-10T purification system (Chengdu Ultrapure Technology Co. Ltd., Chengdu, China) and used throughout all adsorption experiments.

### 4.2. Preparation of Soy Whey Wastewater

Soy whey wastewater was prepared according to the method described by Li et al. [58] with minor adaptations. Briefly, defatted soybean meal was homogenized with tap water at a 1:2.5 (*w*/*w*) ratio in a 10 L glass beaker, and the resulting suspension was maintained at 50 °C. The solution pH was adjusted to 10.0 using an alkaline solution. The mixture was incubated in a DFY-10 thermostatic water bath (Gongyi Yuhua Instrument Co., Ltd., Gongyi, China) at 50 °C under continuous stirring at 1000 rpm for 6 h using a JJ-1B electric stirrer (Changzhou Gaode Instrument Manufacturing Co., Ltd., Changzhou, China). Following extraction, the mixture was centrifuged at 5000 rpm for 10 min in a Sorvall LYNX 6000 centrifuge (Thermo Fisher Scientific, Waltham, MA, USA) to collect the supernatant. The supernatant was subsequently acidified to pH 4.5 and held at 50 °C for 6 h to precipitate soy protein isolate (SPI). The precipitated proteins were removed via a second centrifugation under identical conditions, and the final supernatant was designated as soy whey wastewater and stored at 4 °C until further use.

The composition of the prepared wastewater was quantitatively characterized as follows (mean ± standard deviation): total solids 38.6 ± 3.1 g/L, including total protein 6.8 ± 0.9 g/L, total carbohydrates 21.4 ±1.5 g/L, total isoflavones 1.1 ± 0.2 g/L, and ash content 4.4 ± 0.9 g/L.

### 4.3. Preparation of SWWSAC, SAC, and SA Beads

The SWWSAC composite beads were fabricated via a controlled slow-release cross-linking method. The synthesis procedure was conducted as follows: First, the pH of soy whey wastewater was adjusted to 6.0. Subsequently, 2.0 g of sodium alginate, 1.0 g of cellulose, and 2.0 g of calcium carbonate were dissolved in 100 mL of the pretreated wastewater. The mixture was stirred at 500 rpm and 60 °C for 30 min to achieve complete homogenization. Second, 9 mL of acetic acid was blended with 600 mL of corn oil in a 1000 mL glass beaker and agitated at 200 rpm and 60 °C for 30 min to form a uniformly dispersed oil phase. Third, the alginate–cellulose–wastewater mixture was then slowly injected into the acetic acid–corn oil system using a syringe. The resulting mixture was continuously stirred at 200 rpm and 60 °C for 12 h to facilitate gradual calcium ion release from calcium carbonate and subsequent cross-linking of sodium alginate, leading to bead formation. Fourth, the composite beads were collected using a filter cloth to terminate cross-linking and washed sequentially with dimethyl sulfoxide, absolute ethanol, and deionized water. Each washing step was repeated at least four times to thoroughly remove residual oil and impurities.

For comparative analysis, SAC beads (without soy whey wastewater) and SA beads (without both cellulose and wastewater) were prepared following an identical procedure, using ultrapure water in place of soy whey wastewater. All bead samples were dried at 70 °C and stored in airtight glass containers before further characterization.

### 4.4. Characterizations of SWWSAC, SAC, and SA Beads

The morphological features of SWWSAC, SAC, and SA beads were initially examined using a BC1201E optical microscope (Bosheng Electronic Technology Co., Ltd., Dongguan, China). High-resolution imaging and microstructural analysis were subsequently performed with a Sigma 300 field-emission scanning electron microscope (Zeiss, Oberkochen, Germany), which was equipped with an Xplore 30 energy-dispersive spectrometer (Oxford Instruments, Oxford, UK) for elemental analysis. Specific surface area and pore volume were measured on an ASAP 2000 analyzer (Mike, Norcross, GA, USA) using nitrogen adsorption–desorption isotherms. Surface functional groups of the beads were characterized by Fourier-transform infrared (FTIR) spectroscopy using a Nicolet iS20 spectrometer (Thermo Fisher Scientific, USA). Chemical states of key elements on the SWWSAC bead surfaces, before and after copper (II) ion adsorption, were analyzed via X-ray photoelectron spectroscopy (XPS) on a Nexsa system (Thermo Fisher Scientific, USA). Finally, the point of zero charge (pH_PZC_) of the SWWSAC beads was determined following the pH drift method established by Kosmulski [59].

### 4.5. Measurements of Copper (II) Ion Concentration and Other Concentrations of Cations

The concentration of copper (II) ions was determined colorimetrically using a commercial detection kit (Batch No. 3030377, Zhejiang Lohand Environment Technology Co. Ltd., Hangzhou, China). Absorbance measurements were performed at a wavelength of 560 nm, corresponding to the maximum absorption of the copper (II)-ligand complex. A linear calibration curve was constructed by plotting absorbance (y) against copper (II) ion concentration (x, mg/L), yielding the regression equation y = 0.1127x + 0.0266, with a coefficient of determination (R^2^) of 0.9922. The method demonstrated linearity across the concentration range of 0 to 5.0 mg/L. The concentrations of other cations, including cadmium (II) ion, lead(II)ion, zinc (II)ion, nickel (II)ion, sodium (I) ion, potassium (I) ion and calcium (II) ion were measured by an Avio 220 Max inductively coupled plasma optical emission spectrometer (PerkinElmer, Hopkinton, MA, USA). Before detection, each aqueous solution was centrifuged at 8000 r/min for 10 min and filtered through a 0.22 μm nylon filter membrane to remove suspended particles. Then, the filtrate was acidified to pH < 2 with concentrated nitric acid to prevent adsorption of metal ions onto the walls of the storage container.

### 4.6. Batch Adsorption Experiments for Removal of Copper(II) Ions

Batch adsorption studies were conducted to systematically evaluate the efficiency of copper (II) ion removal from prepared aqueous solutions using SA, SAC, and SWWSAC beads. In each trial, a predetermined mass of beads was added to 250 mL of a copper (II) ion solution in a 500 mL Erlenmeyer flask. The flask was sealed with paraffin film to prevent evaporation and mounted on an SHJ-3 thermostatic magnetic stirrer (Changzhou Danrui Experimental Instrument Co., Ltd., Changzhou, China). The mixture was agitated continuously at 300 rpm and maintained at 25.0 °C. The experiments aimed to systematically examine the influence of key variables on adsorption performance. These included the bead type (SA, SAC, or SWWSAC), initial solution pH (2.0–6.0), SWWSAC dosage (0.1–0.6 g), contact time (10–160 min), and initial copper (II) ion concentration (25–1000 mg/L). The adsorption performance for copper (II) ions was assessed through removal efficiency (*R*_copper_) and adsorption capacity (*q*_copper_), calculated using Equation (1) [14] and Equation (2) [15], respectively.

(1) $$R_{\mathrm{copper}}=(1-\frac{C_{r(\mathrm{copper})}}{C_{o(\mathrm{copper})}})\times 100\%$$

(2) $$q_{\mathrm{copper}}=\frac{(C_{o(\mathrm{copper})}-C_{r(\mathrm{copper})})V}{m}$$

where the initial and residual copper (II) ion concentrations, denoted as *C*_o(copper)_ and *C*_r(copper)_ (mg/L), represent the solution levels before and after adsorption, respectively; *V* is the copper (II) ion solution volume (250 mL), and *m* is the adsorbent mass (0.25 g).

This batch adsorption process was also used for evaluating the removal of copper (II) ion from actual electroplating wastewater, detailed in Table 7, using SWWSAC beads. In the experiments, removal efficiency and adsorption capacity of other cations were calculated based on Equations (1) and (2) with their respective concentrations before and after adsorption. According to these data, adsorption distribution coefficient (*K*_d_) and selectivity coefficient (*K*) were calculated as Equation (3) [60] and Equation (4) [61] for providing a more comprehensive evaluation of the adsorption performance of SWWSAC toward coexisting ions.

(3) $$K_{d}=\frac{(C_{o}-C_{r})V}{C_{o}m}$$

(4) $$K=\frac{K_{d(\mathrm{copper})}}{K_{d(i)}}$$

where *C*_o_ and *C*_r_ are the concentrations of the coexisting ion before and after adsorption, and *K*_d(i)_ is its distribution coefficient.

### 4.7. Adsorption Kinetics, Isotherms and Thermodynamics

To investigate the adsorption kinetics of copper (II) ions onto SWWSAC beads, a series of time-dependent experiments was conducted. The studies were performed at predetermined time intervals (10, 20, 40, 60, 80, 100, 120, 140, and 160 min) under controlled conditions: an initial copper(II) ion concentration of 100 mg/L, an adsorbent dosage of 0.25 g, pH 5.5, and a temperature of 25 °C. Subsequently, the kinetic behavior of the adsorption process was evaluated by fitting the experimental data to three widely recognized models—the pseudo-first-order kinetic (PFOK) model for physisorption, the pseudo-second-order kinetic (PSOK) model for chemisorption and the intraparticle diffusion (ID) model for pore diffusion using the software of Origin Pro 9.0 (Serial number: GF3S4-9089-7991320, OriginLab Corporation, Northampton, MA, USA). The nonlinear forms of these models are provided in Equation (5) [13], Equation (6) [20], and Equation (7) [62], respectively.

(5) $$q_{\mathrm{copper}}=q_{e,\mathrm{copper}}(1-e^{-k_{\mathrm{PFOK}}t})$$

(6) $$q_{\mathrm{copper}}=\frac{k_{\mathrm{PSOK}}{q^{2}}_{e,\mathrm{copper}}t}{1+k_{\mathrm{PSOK}}q_{e,\mathrm{copper}}t}$$

(7) $$q_{\mathrm{copper}}=k_{\mathrm{ID}}t^{0.5}+\theta$$

where *k*_PFOK_ (min^−1^), *k*_PSOK_ (g/(mg·min)) and *k*_ID_ (mg/(g·min^0.5^)) represent the rate constants of the PFOK, PSOK and ID models, respectively; *t* (min) is time; *q*_e,copper_ is equilibrium adsorption capacity of copper (II) ions; θ (mg/g) is the intercept of ID model.

Subsequently, equilibrium isotherm experiments were conducted under a broad range of initial copper (II) ion concentrations, specifically 25, 50, 100, 200, 400, 600, 800, and 1000 mg/L. To evaluate the impact of temperature on adsorption capacity, these experiments maintained consistent operational parameters—including a SWWSAC dosage of 0.25 g, a solution pH of 5.5, and a contact time of 160 min—while varying the temperature to 25 °C, 35 °C, and 45 °C. The obtained equilibrium isotherm data were fitted using two commonly applied models with Origin Pro 9.0 software: the Langmuir adsorption model for monolayer adsorption and the Freundlich adsorption model for multilayer adsorption. The mathematical expressions corresponding to these two models are provided in Equation (8) [15] and Equation (9) [63], respectively.

(8) $$q_{e,\mathrm{copper}}=\frac{q_{\max}K_{L}C_{e,\mathrm{copper}}}{1+K_{L}C_{e,\mathrm{copper}}}$$

(9) $$q_{e,\mathrm{copper}}=K_{F}{C_{e,\mathrm{copper}}}^{1/n}$$

where *C*_e,copper_ (mg/L) stands for the equilibrium concentration of copper (II) ions in the aqueous solution; *q*_e, copper_ (mg/g) denotes the amount of copper (II) ions adsorbed on SWWSAC beads at equilibrium; *q*_max_ (mg/g) refers to the maximum theoretical adsorption capacity of the adsorbent; *K*_L_ (L/mg) is the equilibrium constant associated with the Langmuir model, which reflects the affinity between the adsorbate and adsorbent; and *K*_F_ and 1/n are characteristic constants of the Freundlich model—*K*_F_ is related to the adsorption capacity of the material, while 1/n indicates the sorption intensity.

The thermodynamics of the adsorption of copper (II) ions on SWWSAC beads were analyzed using the Van’t Hoff and Gibbs equations, as described in Equations (10) and (11).

(10) $$\ln K_{d}=-\frac{\Delta H}{RT}+\frac{\Delta S}{R}$$

(11) ΔG=ΔH−TΔS

where *K*_d_ (dimensionless) denotes the adsorption distribution coefficient defined herein as Equation (12) for dimensionless normalization in which m is the used mass of SWWSAC beads, 250 mg and V is the solution volume, 0.25 L [64]; T (in Kelvin, K) represents the operating temperature; ∆G (kJ/mol), ∆H (kJ/mol) and ∆S (J/(mol·K)) correspond to the Gibbs free energy change, enthalpy change, and entropy change, respectively.

(12) $$K_{d}=K_{L}\times \frac{m}{V}$$

### 4.8. Desorption of Copper (II) Ions and Regeneration of SWWSAC Beads

After adsorption reached equilibrium, the SWWSAC beads were collected from the residual copper (II) ion solution and thoroughly washed with ultrapure water. Desorption of the captured copper (II) ions was carried out using 0.1 mol/L HCl as the eluent. To complete the regeneration process, the acid-treated beads were likely neutralized with a dilute NaOH solution and rinsed to remove residual eluent. The regenerated beads were finally applied in consecutive adsorption cycles to assess their stability and recycling potential.

### 4.9. Statistical Analysis

All experimental procedures were conducted in triplicate to guarantee the reproducibility of the findings. Data analysis was performed with Microsoft Excel (Office Professional Plus 2013, Microsoft Corporation, Redmond, WA, USA). For quantifying data variability, results are presented as the mean ± standard deviation. A statistical significance threshold was set at *p* ≤ 0.05 to determine the reliability of the observed differences.

## Figures and Tables

**Table 1:** BET surface areas, pore volumes, and pore sizes of SA, SAC, and SWWSAC beads.

	BET Surface Area (m^2^/g)	Pore Volume (cm^3^/g)	Pore Size (nm)
SA beads	1.18	0.0035	4.03 nm
SAC beads	2.10	0.0031	3.87 nm
SWWSAC beads	3.95	0.021	9.81 nm

**Table 2:** Parameters for fitting the data of adsorption capacity vs. contact time using pseudo-first-order kinetic (PFOK) model, pseudo-second-order kinetic (PSOK) model and intraparticle diffusion (ID) model in Figure 6A.

Models	Parameters	Values
PFOK model	*q*_e,copper_ (mg/g)	65.9 ± 9.0
	*K*_PFOK_ (min^−1^)	13.3
	Adjusted *R*^2^	−0.14286
	*χ* ^2^	732.5
PSOK model	*q*_e,copper_ (mg/g)	112.9 ± 6.4
	*K*_PSOK_ (g/(mg·min))	2.2 × 10^−4^
	Adjusted *R*^2^	0.97149
	χ^2^	18.3
ID model	*K*_ID_ (g/(mg·min^−0.5^))	11.0 ± 0.7
	Θ (mg/g)	−15.9 ± 4.8
	Adjusted *R*^2^	0.98226
	*χ* ^2^	11.9

**Table 3:** Parameters for fitting the data of equilibrium adsorption capacity vs. equilibrium solution concentration using the Langmuir adsorption model and the Freundlich adsorption model in Figure 6B,C.

Temperature (^°^C)	Langmuir Adsorption Model				Freundlich Adsorption Model			
	*K*_L_ (L/mg)	*q*_max_ (mg/g)	Adjusted *R*^2^	*χ* ^2^	*K*_F_ (mg^(1−1/n)^L^1/n^/g)	*n*	Adjusted *R*^2^	*χ* ^2^
25	0.056 ± 0.006	168.3 ± 3.2	0.99272	32.7	38.1 ± 8.5	4.3 ± 0.7	0.92827	322.7
35	0.059 ± 0.007	190.9 ± 4.1	0.99094	54.0	41.6 ± 8.8	4.1 ± 0.6	0.93880	364.6
45	0.062 ± 0.007	204.4 ± 3.9	0.99301	48.6	45.4 ± 9.6	4.2 ± 0.6	0.93734	435.1

**Table 4:** Maximal adsorption capacity of copper (II) ions by sodium alginate-based adsorbents: A comparison of published literature data with results from the present study.

Absorbent	Maximal Adsorption Capacity of Copper (II) Ions (mg/g)	Reference
Nanocellulose/sodium alginate/carboxymethyl-chitosan gel beads	169.9	[14]
Sodium alginate/sodium humate @ Polyacrylamide hydrogel	134.7	[45]
Sodium alginate-Mg(OH)_2_ hydrogels	253.37	[46]
Glutaraldehyde- humic acid/sodium alginate composite membrane	63.1	[47]
Sodium alginate-melamine sponge composites	90.1	[48]
Fe- graphene oxide/montmorillonite/sodium alginate composites	116.4	[49]
ZIF-8@ sodium alginate composite hydrogels	309.6	[50]
MXene/polyaniline/sodium alginate composite gel	255.8	[51]
Zr^4+^-GMSA aerogel	126.7	[52]
Chitosan/carboxymethyl cellulose/sodium alginate magnetic composite hydrogels	224.49	[53]
SWWSAC beads	168.3–204.4	Current work

**Table 5:** Calculated thermodynamic parameters for the adsorption of copper (II) ions onto SWWSAC beads.

Temperature (K)	∆H (kJ/mol)	∆S (J/(mol·K))	∆G (kJ/mol)
298.15	4.0	46.9	−10.0
308.15			−10.5
318.15			−10.9

**Table 6:** Parameters for various cations adsorbed at SWWSAC beads from actual electroplating wastewater.

Component	Initial Concentration, *C*_o_ (mg/L)	Final Concentration, *C*_r_ (mg/L)	Removal Efficiency, R (%)	Adsorption Capacity, q (mg/g)	Distribution Coefficient, *K*_d_ (L/g)	Selectivity Coefficient, *K*
Copper (II) ion	203	18.3 ± 0.7	92.4 ± 0.4	57.7 ± 0.2	3.15	/
Cadmium (II) ion	3.1	2.67 ± 0.05	13.7 ± 0.6	0.13 ± 0.01	0.05	63.0
Lead (II) ion	0.5	0.37 ± 0.01	26.0 ± 2.9	0.04	0.11	28.6
Zinc (II) ion	8.4	6.9 ± 0.05	17.5 ± 0.3	0.46 ± 0.01	0.07	45.0
Nickel (II) ion	1.2	0.93 ± 0.03	21.9 ± 2.1	0.08 ± 0.01	0.09	35.0
Sodium (I) ion	286	264.4 ± 7.3	7.6 ± 0.2	6.75 ± 0.17	0.03	105
Potassium (I) ion	29	26.5 ± 1.8	8.6 ± 0.3	0.78 ± 0.03	0.03	105
Calcium (II) ion	58	33.6 ± 1.7	42.1 ± 1.2	7.6 ± 0.2	0.22	14.3
pH	5.5	5.1 ± 0.1	/			

**Table 7:** Composition of actual electroplating wastewater.

Component	Concentration (mg/L)
Copper (II) ion	203
Cadmium (II) ion	3.1
Lead (II) ion	0.5
Zinc (II) ion	8.4
Nickel (II) ion	1.2
Sodium (I) ion	286
Potassium(I) ion	29
Calcium (II) ion	58
Sulfate (II) ion	634
Chloride (I) ion	158
Ammonia nitrogen	43.6
Nitrate nitrogen	98.5
Total phosphorus	10.4
Dissolved organic carbon	68
Suspended solids	168
pH	4.9

## Data Availability

The data are contained within the article.

## Supplementary Materials

The following supporting information can be downloaded at: https://www.mdpi.com/article/10.3390/gels12060464/s1. Figure S1. Distribution of aqueous copper species as a function of pH at a copper(II) ion concentration of 200 mg/L. (The data was obtained using the software of Visual MINTEQ 4.0 (Jon Petter Gustafsson at the Swedish University of Agricultural Sciences (SLU), Uppsala, Sweden)). Figure S2. FTIR spectra of SWWSAC beads before copper (II) ion adsorption and after the 6th recycling cycle.
