# Supplementary material for: Soy Whey Wastewater-Derived Sodium Alginate/Cellulose Composite Beads for Efficient Copper (II) Ion Adsorption: Performance and Mechanism
Source: Gels. 2026 May 26;12(6):464. doi: 10.3390/gels12060464 (PMC13297876; doi:10.3390/gels12060464)
Supplement: Supplementary file 1 [file gels-12-00464-s001.zip › gels-4306521-supplementary.pdf]

Supplementary Materials

Soy whey wastewater-derived sodium alginate/cellulose com-posite beads for efficient copper(II) ion adsorption: Perfor-mance and mechanism

Rui Li *a, Chang Xu a, Qiannuo Gu a, Xiaoyang Pan a, Andong Qian a and Xuning Leng *b

[a]School of Life Sciences, Jining Medical University, No. 669 Xueyuan Road, Donggang District, Rizhao, Shan-dong Province, 276826, China

[b]Municipal Science and Technology Innovation Service Center, Jining Road No.369, Donggang District, Rizhao City, 276800, Shandong, P. R. China

*Correspondence: ruili061289@163.com (Rui Li); lxning1114@163.com (Xuning, Leng)

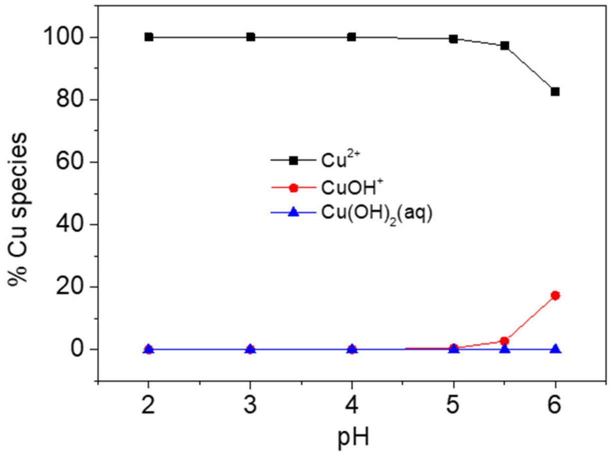

Figure S1 Distribution of aqueous copper species as a function of pH at a copper(II) ion concentration of 200 mg/L. (The data was obtained using the software of Visual MINTEQ 4.0 (Jon Petter Gustafsson at the Swedish University of Agricultural Sciences (SLU), Uppsala, Sweden.))

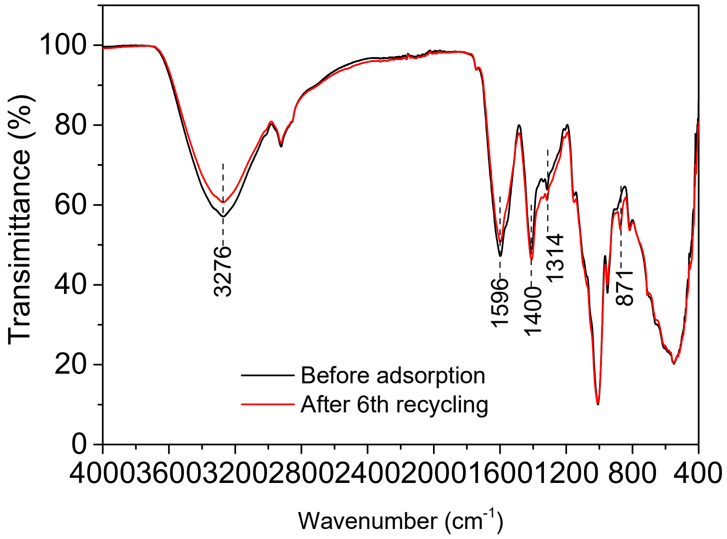

Figure S2 FTIR spectra of SWWSAC beads before copper(II) ion adsorption and after the 6th recycling cycle.
